# Human V α 7.2-J α 33 mucosal-associated invariant T cells in endometrial ectopic tissues tend to produce interferon-gamma: A new player in endometriosis etiology: A case-control study

**DOI:** 10.18502/ijrm.v22i3.16168

**Published:** 2024-05-15

**Authors:** Maryam Zare Moghaddam, Fateme Zare, Reyhane Sandoghsaz, Abbas Khalili, Ali Shams

**Affiliations:** ^1^Department of Immunology, Shahid Sadoughi University of Medical Sciences, Yazd, Iran.; ^2^Reproductive Immunology Research Center, Shahid Sadoughi University of Medical Sciences, Yazd, Iran.; ^3^Abortion Research Center, Yazd Reproductive Sciences Institute, Shahid Sadoughi University of Medical Sciences, Yazd, Iran.; ^4^Department of Pediatrics, Shahid Sadoughi Hospital, Shahid Sadoughi University of Medical Sciences, Yazd, Iran.

**Keywords:** Endometriosis, MAIT, IFN-γ, TNF-α, TCR V alpha 7.2-J alpha33, IL-17.

## Abstract

**Background:**

Endometriosis is a chronic estrogen-related inflammatory disorder that is known by proliferating endometrial cells in a place outside the uterus. The high presence of immune cells in the peritoneal fluid of women with endometriosis confirms the involvement of the immune system in the pathogenesis of the disease. Mucosal-associated invariant T (MAIT) cells play an undeniable impact on mucosal immunity by the production of interleukin-17, interferon-gamma (IFN-γ), and tumor necrosis factor-alpha. The function of the cells in the pathogenesis of endometriosis is less investigated.

**Objective:**

This study aims to investigate the infiltration of MAIT cells by using the determination levels of *V α 7.2-J α 33* gene expression in eutopic and ectopic tissue of endometriosis lesions.

**Materials and Methods:**

In this case-control study, the tested samples include 20 eutopic and 20 ectopic tissues of women with endometriosis and 20 uterine endometrial tissues of women in the control group. Expressions of the *V α 7.2-J α 33* tumor necrosis factor-alpha, interleukin-17A, and *IFN*-γ genes were analyzed by quantitative reverse transcriptase-polymerase chain reaction.

**Results:**

According to the results, *V α 7.2-J α 33* gene expression did not show substantial elevation in the uterine and eutopic endometrial tissues compared to internal gene control as well as in ectopic tissues. Correlation analysis approved a positive relationship between *V α 7.2-J α 33* expression genes and *IFN*-γ levels in ectopic tissues.

**Conclusion:**

Considering the low-expression specific gene of MAIT cells in ectopic tissue, it can be concluded that these cells are present in the endometriotic environment to a certain extent, and there is a possibility of their role in the progression of endometriosis by secreting IFN- γ .

## 1. Introduction

Endometriosis is a chronic inflammatory condition characterized by endometrium-like tissue proliferation and development outside the uterus. The main symptoms of the condition are pain, infertility, adenomyosis, inflammation, and alterations in the placentation (1). “Endometriosis affects 10–15% of all women of reproductive age and 70% of women with chronic pelvic pain" (2). Despite several investigations, many concerns about endometriosis pathogenesis remain unanswered. Endometriosis is caused by a combination of variables, including genetics, immunological and hormonal characteristics, and prostaglandin metabolism (3).

Previous research has found alterations in the frequency and function of immune cells in endometriosis participants' peripheral blood, peritoneal fluid, and endometriotic tissue, which supports the involvement of immune responses in this disease (4). Although endometriosis is an inflammatory disease, immune-suppressing cells like M2 macrophages (type 2 macrophage), regulatory T cells, helper T cells type 2, and myeloid-derived suppressor cells, as well as related cytokines like interleukin (IL)-10 and transforming growth factor beta, have all been linked to disease progression (5–7). Mucosal-associated invariant T (MAIT) cells are a type of T cell with a non-variable alpha chain receptor known as V α 7.2-J α 33 and a limited diversity beta chain restricted to a non-polymorphic major histocompatibility complex I known as MR-1 in humans. These cells have a variety of surface markers, including CD3, CD25, CD8 αα , CCR25, CCR6, CCR9, and CXCR6, CD122, CD95, CD69, CD27, and CD161, and their number varies according to the tissue. Their prevalence in the liver and the intestinal lamina propria is 20–50% and 1–10%, respectively. MAIT cells in humans are activated by the specific identification of bacterial and fungal antigens (including vitamin B2 and B9 metabolites) on MR-1 molecules. MAIT cells also express IL-18 and IL-12R permanently, resulting in cell activation by IL-12 or IL-18 (8). According to recent findings, MAIT cells have a role in mucosal immune response and the maintenance of lung and gut mucosal tissues. Bister and colleagues demonstrated that MAIT cells persist in the endometrium and decidua, resulting in powerful defense responses against Neisseria gonorrhoeae. Furthermore, the existence of MAIT cells in vaginal mucous and their function are allegedly dependent on Th17 and the IL-17/IL-22 axis. MAIT cell frequency rises in infected and inflamed tissues, either by blood movement or local proliferation at infected locations (9–12).

Most findings have supported enhanced inflammation in ectopic lesions, although there is very little information on MAIT cells in the development of inflammation in endometriosis lesions, as well as the cells' potential to produce IL-17, tumor necrosis factor-alpha (TNF- α ), and interferon-gamma (IFN- γ ) in the tissues. Given the importance of MAIT cells in mucosa-dependent immunity and lack of understanding of their role in endometriosis, this study intends to evaluate the cells role in the illness. This study aims to gain more knowledge in the field of MAIT cells in mucosal immune defense, the pathogenesis of inflammatory diseases as well as cancer, as well as the lack of sufficient information about the role of this innate immune cell in endometriosis.

## 2. Materials and Methods

### Participant population 

Sample collection for this case-control study was performed in Obstetrics and Gynecology Departments of Shahid Sadoughi hospitals and sample analysis was conducted in Reproductive Immunology Research Center, Shahid Sadoughi University of Medical Sciences, Yazd, Iran from May 2021 to September 2022. 20 endometriosis women with stage III, IV were discernment through laparoscopy and 20 eutopic and ectopic tissue was prepared. As a control group, 20 healthy women who underwent laparoscopy for diagnostic reasons, tubal disconnection, ovarian cystectomy, ovarian drilling were included in the study. Women who had previously received hormonal medications, immunosuppressive drugs, or had a history of autoimmune, cancer, or infectious disorders were excluded from participating in the research. Before the start of the study, all participants were asked to fill out an informed consent form.

### Gene expression in endometriotic tissue

#### Samples collection

The specimen tissues that were resected by laparoscopy method were 50–100 mg, they were later frozen in the RNA solution and were kept at -80 C until extraction of mRNA to preserve their RNA stability.

#### mRNA extractions

Homogenization was carried out using a sterile syringe and needle. Cell and tissue lysates were passed through a 20-gauge (0.9 mm) needle at least 5–10 times. TRIPURE Isolation Reagent kit (Gene-Foci, Switzerland) was used for RNA extraction and this process was carried out according to the kit instructions. Concentration, purity, and quality of extracted RNA were defined using spectrophotometric analysis (Thermo Fisher NanoDrop). All the disposal equipment used during the RNA extraction process was RNAase free.

#### Polymerase chain reaction (PCR) method

cDNA was synthesized of template mRNA according to the kit's instructions. The PCR reactions were carried out in a 20 µl total mixture volume including: 500 ng RNA, 10 µL buffer-mix (2x), 2 µl enzyme, and 8 µl of DEPC-treated. PCRs cycles were started by incubating the mixture for 4 min at 95 C, followed by 35 cycles composed of 94 C for 30 sec, 57 C for 30 sec, 72 C for 30 sec, and 72 C for 5 min and product size was confirmed on 1% agarose gel.

#### Quantitative real-time PCR method

The SYBER green PCR master mix was used in accordance with the kit protocol, and the mRNA expression levels were evaluated using the ABI/PRISM 7000 sequence detection system (Applied Biosystems, Foster, CA). Thermal cycling settings included 5 min at 95 C, followed by 40 cycles of 15 sec at 95 C, 60 sec at 61 C, and 45 sec at 72 C. Beacon designer 7.9 software was used to design specific primers for amplification of desired genes in the form of exon junction (in this method, one of the primers is designed exactly on the junction of exons) and intron inclusion (in this method, an intron with a length of at least 300 bp was performed). Table I provided the primer V α 7.2-J α 33 (13).

**Table 1:** Sequence of primers


**Genes**	**Sequence (5 '→ 3 ' )**	**Mer**	**PCR product length**
	F: GTCGGTCTAAAGGGTACA	18	
***V α 7.2-J α 33***	R: CCAGATTAACTGATAGTTGCTA	22	104bp
		F: TTTTAATGCAGGTCATTCAGATGT		22	
***IFN- α ***	R: AAGTTTGAAGTAAAAGGAGACAATTTGG	20	220bp
		F: CATGAACTCTGTCCCCATCC		22	
***IL-17A***	R: CCCACGGACACCAGTATCTT	20	102bp
		F: ACCTCAGGGCTAAGAGCGCA		20	
***TNF- α ***	R: CTGACTGCCTGGGCCAGAGG	20	143bp
		F: AAGATGAGTATGCCTGCCGTG		21	
*** α 2Microglobulin***	R: CGGCATCTTCAAACCTCCAT	20	105bp
	PCR: Polymerase chain reaction, *IFN*-γ: Interferon gamma, *TNF- α *: Tumor necrosis factor α , *IL-17A*: Interleukin-17			

### Ethical considerations 

Each participant provided written informed consent before participating in the study. This research was supported by a grant from Research Ethics Committees of Shahid Sadoughi University of Sciences, Yazd, Iran (Code: IR.SSU.MEDICINE.REC.1400.097).

### Statistical analysis

Statistical package for social science version 25 was used for statistical analysis. For normality distribution Shapiro-Wilk and Kolmogorov-Smirnov tests were performed, where they found that the data were not normally distributed by p < 0.05. So, the Mann-Whitney U-test was used for the comparison of *IFN*-γ, *IL-17-A*, *TNF- α ,* and *V α 7.2-J α 33* levels in each group against the control group. The Kruskal-Wallis test was used to compare the analytes in all groups. Also, we repeated measures of tests for the evaluation of the analytes at different times. The results are expressed as mean ± standard error of the mean. Any p < 0.05 was considered to be statistically significant. All graphs were drawn using GraphPad Prism 5 (Graphpad Software Company, USA).

## 3. Results

### Demographic characteristics of the studied subjects

In this study, the average age and weight of women in the control group were 41 yr and 71 kg, respectively, while of those in the patient group were 35 yr and 64 kg, respectively.

### Quality control results of synthesized cDNA

**Figure 1:**
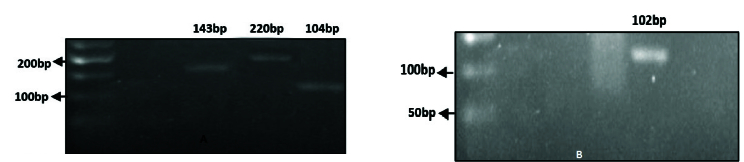
Results of PCR product electrophoresis, 1% agarose gel was used for cDNA electrophoresis. A) Include bands of V α 7.2-J α 33 with a length of 104 bp and IFN- γ with a length of 220 bp. B) Include bands of TNF- α with a length of 143 bp, and IL-17A with a length of 102 bp.

**Figure 2:**
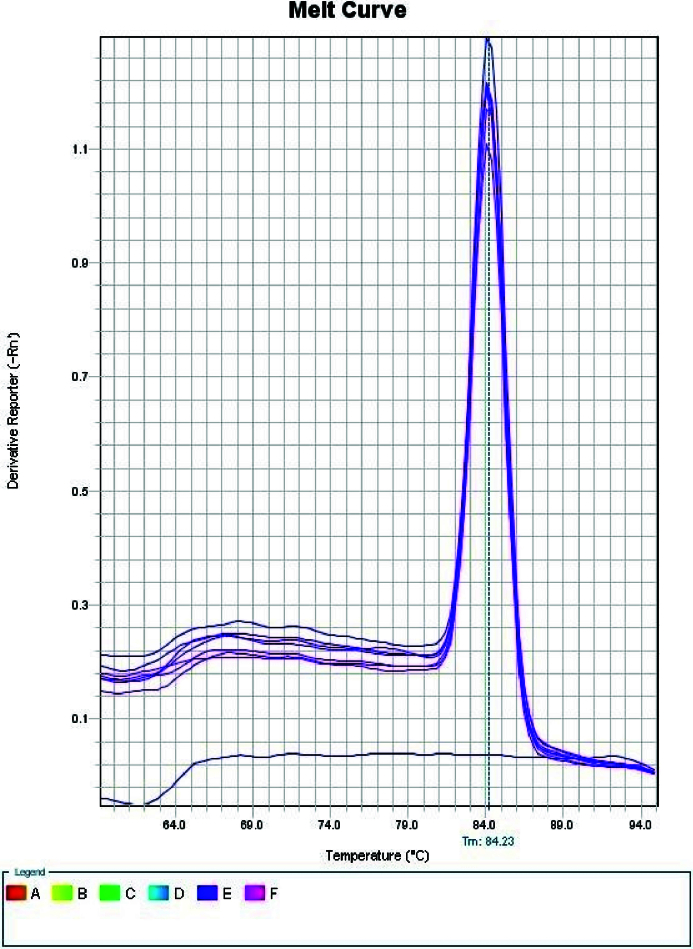
*IFN*-γ melt curve.

The electrophoresis technique was used to assess the quality of the cDNA produced. Following cDNA synthesis, cDNA-containing samples were run on an agarose gel, yielding bands V α 7.2-J α 33 with a length of 104 bp, IFN- γ with a length of 220 bp, TNF- α with a length of 143 bp, IL-17A with a length of 102 bp (Figure 1), and melt curve of IFN- γ was observed in figure 2.

### Real-time PCR results

#### Comparison of *IFN-γ* gene expression in endometriosis participants with control group

**Figure 3:**
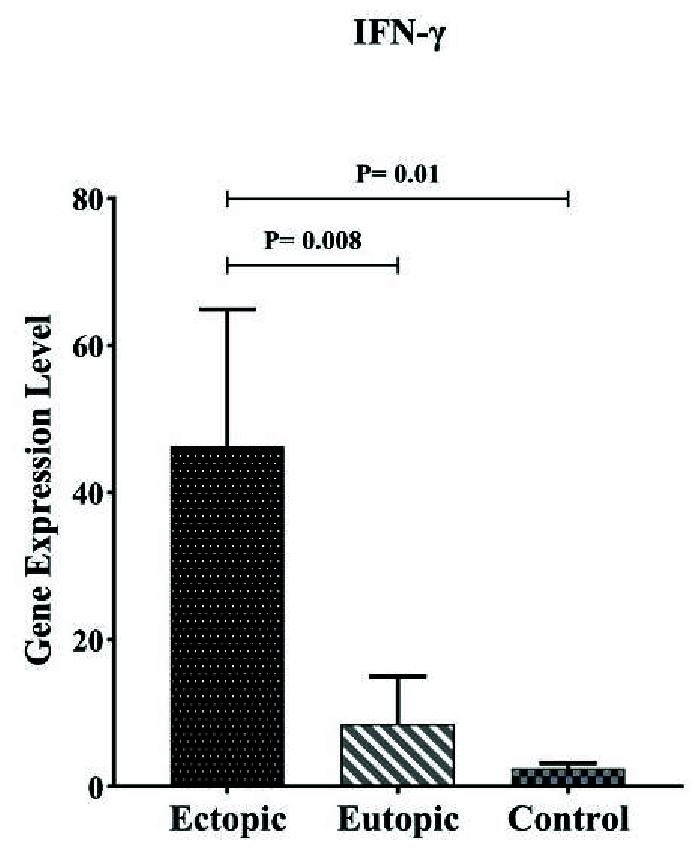
Expression of *IFN*-γ mRNA in ectopic, eutopic, and healthy women. Statistical analysis showed significant elevation levels of *IFN*-γ in ectopic lesions compared to the eutopic lesion and control females.

Table II shows that the expression of *IFN*-γ in ectopic tissue was significantly higher than in eutopic endometrium and normal endometrium, with p = 0.008 and p = 0.01 respectively. Furthermore, the expression of this gene in eutopic tissue does not differ significantly from the control group (Table II, Figure 3).

#### Comparison of *TNF-α* gene expression in endometriosis participants with control group

**Figure 4:**
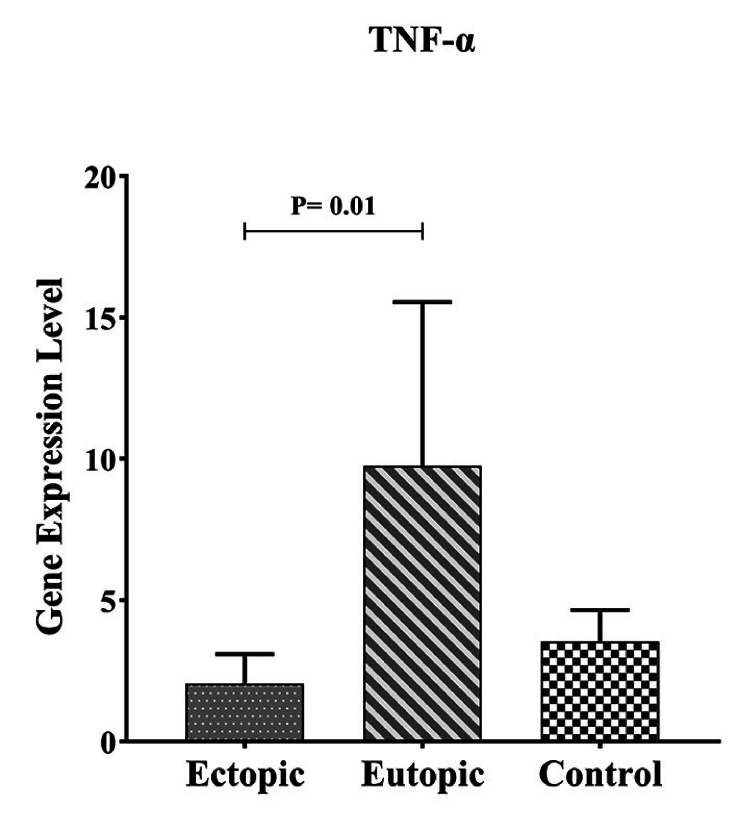
Expression *TNF- α* mRNA in the endometriotic lesion and normal endometrium. Higher level of *TNF- α* in eutopic lesions compared to ectopic lesions in endometriosis participants with p = 0.01.

As shown in figure 3, the expression of *TNF- α* in eutopic tissue was significantly higher than in ectopic tissue with p = 0.01 (Table II, Figure 4).

#### Comparison of *IL-17A* gene expression in endometriosis participants with control group

**Figure 5:**
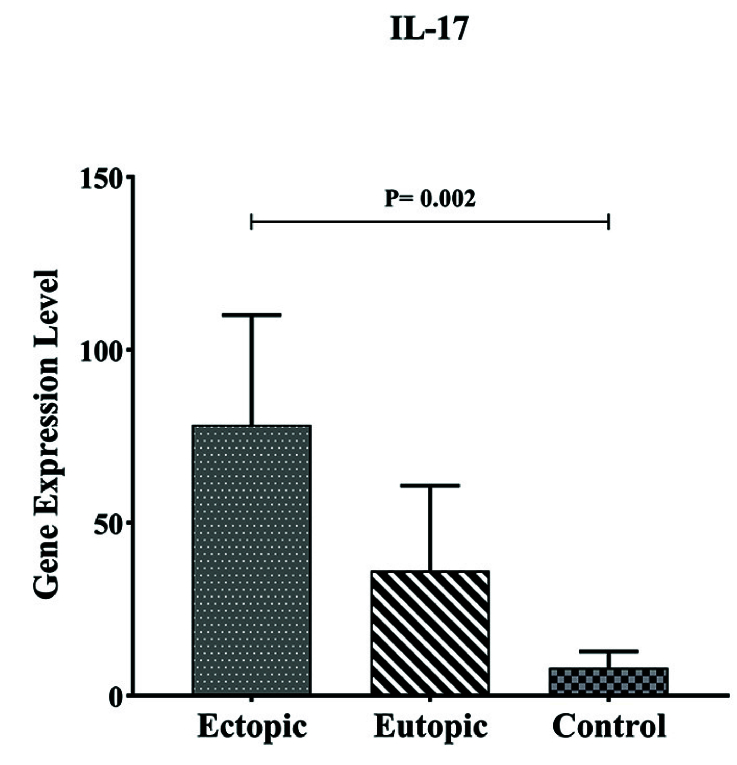
Expression *IL-17* mRNA in the endometriotic lesion and normal endometrium. A higher level of *IL-17 *in the ectopic lesion was observed compared to the eutopic lesion and control with p = 0.002.

Our results demonstrated that *IL-17A *expression in ectopic tissue was considerably higher than in eutopic and control groups, with p = 0.002 (Table II, Figure 5).

#### The results of examining the expression of *Vα7.2 Jα33* gene in ectopic, eutopic, and control tissue

**Figure 6:**
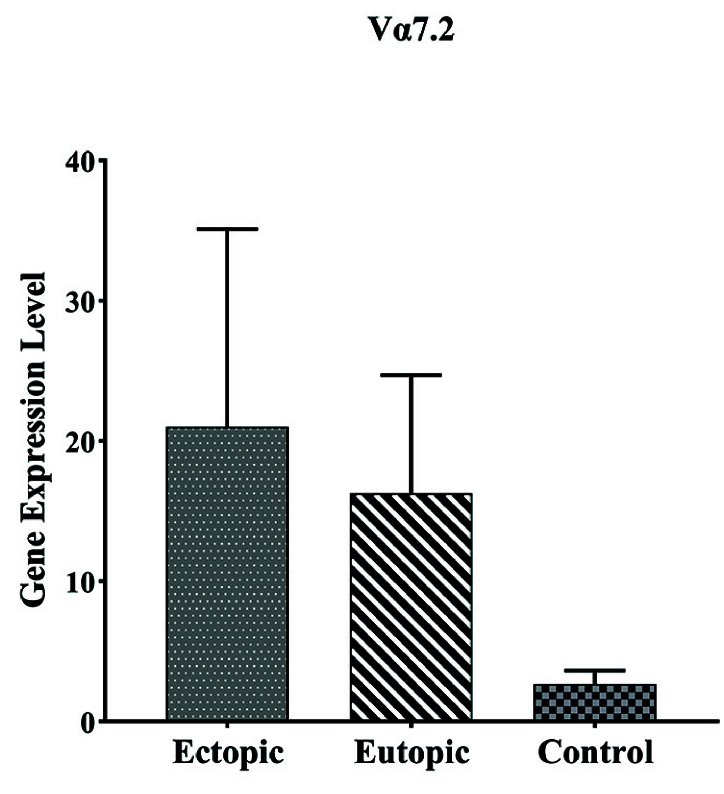
Expression *V α 7.2-J α 33* mRNA in the endometriotic lesion and normal endometrium. No statistically significant difference was observed in *V α 7.2-J α 33* mRNA expression in endometriotic lesions compared to control.

As indicated in table III, *V α 7.2-J α 33* mRNA was expressed in all samples, and no significant difference was observed in expression between ectopic and eutopic tissue, with p = 0.54. Furthermore, the expression of this gene in ectopic tissue was higher than in the control group but it is not significant with p = 0.16 (Table II, Figure 6).

#### The results of investigating the correlation between the expression levels of *Vα7.2-Jα33* and *IFN-γ*, and *TNF-α* and *IL-17*

The correlation test results show a positive relationship between *V α 7.2 J α 33* and *IFN*-γ expression levels in endometriotic tissue from endometriosis participants (p: 0.01, r: 0.404).

**Table 2:** Expression levels of *IFN- γ , TNF- α , IL-17, V α 7.2-J α 33* mRNA in the study groups


**Variables**	**Ectopic group ${\;}^{\text{a}}$ **	**Eutopic group ${\;}^{\text{b}}$ **	**Control group ${\;}^{\text{c}}$ **	**P-value**
***IFN*** **-γ**	46.25 ± 18.65 10.28 (0.49–59.51)	8.52 ± 6.46 0.58 (0.12–2.19)	2.47 ± 0.71 1.04 (0.30–3.95)	0.01 a, b: 0.008 a, c: 0.01 b, c: 0.34
***TNF-α***	2.05 ± 1.03 0.47 (0.05–1.60)	9.75 ± 5.79 1.69 (0.96–8.22)	3.54 ± 1.11 2.01 (0.19–3.81)	0.04 a, b: 0.01 a, c: 0.08 b, c: 0.64
***IL-17***	78.33 ± 31.78 11.22 (2.34–119.2)	36.11 ± 24.65 4.09 (0.01–8.92)	8.10 ± 4.73 1.39 (0.49–5.12)	0.01 a, b: 0.06 a, c: 0.002 b, c: 0.32
***Vα7.2-Jα33***	21.01 ± 14.10 1.99 (0.55–10.37)	16.27 ± 8.43 2.32 (0.20–17.49)	2.67 ± 0.96 0.65 (0.36–3.76)	0.42 a, b: 0.54 a, c: 0.16 b, c: 0.62
Data expressed as Mean ± SEM (Median [IQR]), ANOVA followed by Tukey's and *t* test. *IFN*-γ: Interferon-gamma, *TNF- α *: Tumor necrosis factor α , *IL-17*: Interleukin-17, P: Probability, a: Ectopic group, b: Eutopic group, c: Control group				

**Table 3:** The correlation analysis between the expression levels of *V α 7.2-J α 33, IFN- γ , TNF- α , *and* IL-17* gene


**Variables**	***Vα7.2-Jα33***	***IFN*** **-γ**	***TNF-α***	***IL-17***
***Vα7.2-Jα33***	r: 1.000 P: 0	r: 0.404 P: 0.01	r: -0.050 P: 0.76	r: 0.083 P: 0.65
***IFN*** **-γ**	r: 0.404 P: 0.01	r: 1.000 P: 0	r: 0.102 P: 0.53	r: -0.094 P: 0.61
***TNF-α***	r: -0.050 P: 0.76	r: 0.102 P: 0.53	r: 1.000 P: 0	r: -0.063 P: 0.73
***IL-17***	r: 0.083 P: 0.65	r: -0.094 P: 0.61	r: -0.063 P: 0.73	r: 1.000 P: 0
The correlation between variables was examined with the Pearson correlation test. r: Correlation coefficient, P: P-value, *IFN*-γ: Interferon-gamma, *TNF- α *: Tumor necrosis factor α , *IL-17*: Interleukin-17				

## 4. Discussion

Despite numerous efforts to determine the etiology of endometriosis, the exact role of immune system components, especially MAIT cells, remains unknown. Based on previous findings in ovarian endometriotic a large number of immune cells were found in the lesions, but the majority of these cells had an immunosuppressive phenotype, implying defects in immune surveillance functions caused by a suboptimal immune response, which led to the development of endometriosis (13).

Recently, based on our increased understanding of MAIT cell potential in inflammatory responses and ample proof of the cells' existence in female vaginal mucosal organs, the cells have become suspects in infertility and reproduction diseases. Not only do these cells have direct impacts on bacterial infection immunology, but they also play an important role in the progression of various mucosal-related cancers, such as colorectal cancer. Our findings reveal that MAIT cell infiltration into grade III and IV endometriotic lesions is minimal, but we approve of the cell's presence in ectopic and utopic ovarian endometriosis environments. Also, with the increase in the sample size, there is a possibility that the difference between the ectopic group and the control group will reach a significant level. A low-level of V α 7.2-J α 33 MAIT cells are present in uterine tissues, while the influx of CD8-positive MAIT cells in peritoneal fluid of endometriosis participants was significantly elevated (14).

Related previous research found that lymphocytes in endometriosis tissue have a higher capacity for IFN- γ production than cells in eutopic tissues (15). MAIT cells in the uterus have an active phenotype and a high potential for IFN- γ production. Our findings revealed a substantial increase in the expression of IFN-γ mRNA in ectopic tissue as compared to eutopic and control groups. More importantly, correlation analysis indicated that IFN-γ expression levels have a positive connection with V α 7.2-J α 33 gene expression, implying that MAIT cells may be a source of the cytokine in ectopic lesions. An in vitro study discovered that combining IFN- γ and IL-10 can increase ectopic cell proliferation and penetration (16).

As a result, MAIT cells play a role in the development of endometriosis by generating IFN- γ . According to these data, although the frequency of MAIT cells in the endometrium is low, their potential to produce cytokines can induce inflammatory responses. Also IFN- γ generating MAIT cell play role in pathogenesis of lung cancer and type 1 diabetes through increasing inflammation (17, 18).

Other studies defined increase and pathogenic role of this cytokine in peritoneal fluid and eutopic endometrium of participant with endometriosis through increase inflammation and up-regulate the expression of sICAM-1 in eutopic endometrium and interfere with immune function by ICAM-1, respectively (19, 20).

Our findings further confirmed that IL-17A gene expression increased in eutopic and ectopic endometriosis tissue as compared to control groups. T helper-17 and ectopic lesion in endometriotic tissue producing IL-17 can lead to progression of endometriosis through increasing inflammation (4). Our statistical analysis did not reveal a correlation between elevated IL-17A and V α 7.2-J α 33, indicating that the cytokine's source is not MAIT cells in the tissues. The potential of MAIT cells to produce IL-17 has already been proven in numerous autoimmune illnesses and immunological disorders. Despite IL-17 conventional role in inflammation, newly discovered angiogenic characteristics of the cytokine may increase endometriotic cell growth (21). MAIT, Th17, and IL-17, when combined, can cause a pro-inflammatory response. However, the immunosuppressive action of MAIT cells has been found in some instances (14, 22). Interestingly, MAIT cells, due to their potential to develop myeloid-derived suppressor cells and neutrophils, may promote tumor growth and carcinogenesis (23).

Even though TNF- α was found to be highly expressed in eutopic tissue, correlation analysis revealed no significant relationship with V α 7.2-J α 33 gene expression. In other studies, TNF- α expression was found to be significantly higher in ectopic endometriosis tissue than in eutopic tissue (24). Inconsistently, in another study, TNF- α gene expression did not show significant differences in ectopic ovarian tissue and endometrial uterine tissues (25).

High expression of t-bet (specific transcription factor of T helper-1) in eutopic tissue show high existence of T helper-1 in eutopic tissue which can produce TNF in this place (26). According to new research, TNF- α also decreases the expression of miR-183 in eutopic and ectopic tissue, leading to increased endometriotic cell invasion and proliferation (27). TNF- α also promotes angiogenesis by raising the expression of prokineticin-1 in eutopic tissue (28). In inflammatory situations, progesterone receptors are downregulated under effect of TNF- α , and progesterone resistance develops, leading to endometriosis (29). Furthermore, TNF- α by inducing PGE2 in stromal cells of eutopic tissue, promotes the progression of endometriosis (30).

However, to achieve a more detailed understanding of the role of MAIT cells in this disease; examining the subgroups of this cell (CD4+, CD8+, and CD4/CD8-/- [double negative, DN]), determining their frequency and activity with CD marker in both the initial and final stages of the disease, and also increasing the number of participants for further studies were put on the agenda.

## 5. Conclusion

MAIT cells, which are innate immune cells, are found in low numbers in the ectopic ovarian tissue of endometriosis participants and may play a role in disease pathogenesis by secreting IFN- γ .

##  Data availability

Data supporting the findings of this study are available upon reasonable request from the corresponding author.

##  Author contributions

Ali Shams and Abbas Khalili designed the study and conducted the research. Fateme Zare and Maryam Zare Moghadam and Reyhane Sandoghsaz evaluated essential criteria for selecting patients and carried out the experiments and analyzed the result of the study. All authors approved the final manuscript and take responsibility for the integrity of the data.

##  Conflict of Interest

The authors declare that there is no conflict of interest.
